# Seroprevalence of SARS-Cov-2 in the setting of a non-dedicated COVID-19 hospital in a low CoV-2 incidence area: Implications for surgery

**DOI:** 10.1016/j.amsu.2020.11.006

**Published:** 2020-11-03

**Authors:** Fabio Medas, Federico Cappellacci, Giacomo Anedda, Gian Luigi Canu, Stefano Del Giacco, Pietro Giorgio Calò, Davide Firinu

**Affiliations:** a Department of Surgical Sciences, University of Cagliari, Cagliari, Italy; b Department of Medical Sciences and Public Health, University of Cagliari, Italy

## Abstract

The aim of this study was to assess the seroprevalence of SARS-Cov-2 in the setting of a non-dedicated COVID-19 hospital in a low CoV-2 incidence area. We analysed the data of the patients admitted at our surgical department during the period 31st March - June 30, 2020. Among 86 patients included in the study, we found 2 (2.3%) patients positive for both SARS-CoV-2 specific IgM and IgG, 2 (2.3%) for only SARS-CoV-2 specific IgM, and 1 for only SARS-CoV-2 specific IgG. Thus, seroprevalence for SARS-CoV-2 was 5.8%; nasopharyngeal swab was negative in all the cases. Considering the current limitations in sensitivity of nasopharyngeal swab, the uncertainty in the natural history of SARS-CoV2, and the reported prevalence of CoV-2, we think that careful preadmission triage and tests, the use of personal protective equipment and safe management of surgical smoke are mandatory also in our context of low CoV-2 incidence area.

Italy has been hit hard by the COVID-19 pandemic, with almost 150,000 confirmed cases and 35,000 deaths, placing an extreme burden on healthcare systems worldwide [[1], [2], [3]]. Nevertheless, in our region, Sardinia, the incidence of COVID-19 has been relatively low, with almost 1500 cases and 150 deaths on a population of 1.6 million people. On 10th March, Italian government imposed a national quarantine, and the activity of hospitals were reorganized to deal with the emergency.

In our Department of General and Endocrine Surgery of University Hospital of Cagliari, a non-dedicated COVID-19 hospital, following the indications of the Italian Ministry of Health, surgical activity was reduced from mid-March, operating mainly urgent and oncologic patients.

We have herein analysed the patients admitted to our Department that underwent serologic tests for SARS-CoV-2 either by Antibodies (Ab) or Reverse Transcriptase-Polymerase Chain Reaction (RT-PCR), to estimate the prevalence of COVID-19 in the setting of a non-dedicated COVID-19 hospital and in a low CoV-2 incidence area (2% in preliminary estimates for Sardinia), and to evaluate if security measures are necessary and adequate in this context.

During the study period (31st March – June 30, 2020), 129 patients were admitted to our Department; among these, 86 (66.6%) patients performed serologic tests for SARS-CoV-2 and were the object of this study. A chemiluminescent analytical system (CLIA) for the detection of both IgM and IgG antibodies against SARS-Cov-2 S-antigen and N-protein on Maglumi platform (Snibe, Shenzhen, China) was employed (IgM cut-off is 1.0 AU/mL, while the IgG cut-off is 1.1 AU/mL). Thirty-two (39.5%) patients were males and 54 (60.5%) females, with a mean age of 57.6 years. Seventy-six (88.4%) patients were admitted for planned surgery, whereas 10 (11.6%) patients were transferred from Emergency Department to our Surgical Unit. The main indication for elective surgery was malignancy in 42 (55.3%) cases, including thyroid, breast, colorectal, and skin cancer.

Before admission to our ward, patients were evaluated for SARS-CoV-2 risk factors, including symptoms during the last 2 weeks and close contact with confirmed cases. All personnel involved in pre-admission tests used personal protective equipment (PPE), including goggles, gowns, gloves, and caps. Two (2.3%) patients had fever at admission and 2 (2.3%) patients had a confirmed-case relative; no other relevant symptoms were found. A chest CT scan was performed in 62 (72.1%) patients, and a bilateral interstitial involvement suggestive for COVID-19 was found in 3 cases. At serology, 2 (2.3%) patients tested positive for both SARS-CoV-2 specific IgM and IgG (one of them had a suspicious CT scan), 2 (2.3%) for only SARS-CoV-2 specific IgM, and 1 for only SARS-CoV-2 specific IgG (Fig. 1). Thus, seroprevalence for SARS-CoV-2 was 5.8%. All the patients underwent at least one nasopharyngeal swab before admission to our department, which was negative in all the cases.

**Fig. 1:**
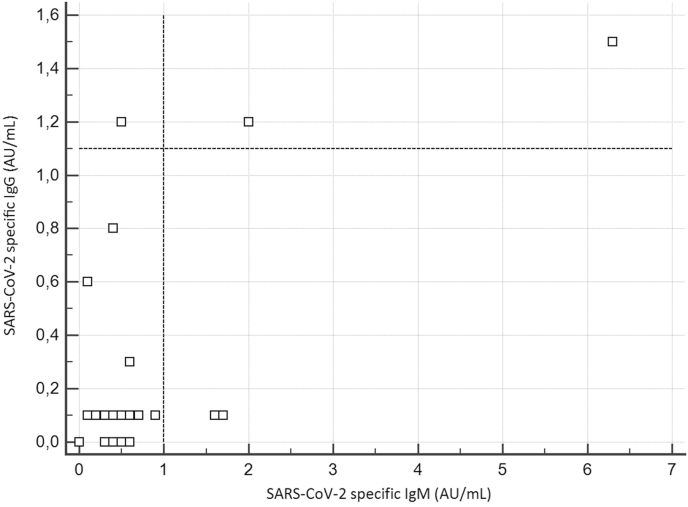
SARS-CoV-2 Antibodies testing. Each square represents a patient. IgM cut-off is 1.0 AU/mL, IgG cut-off is 1.1 AU/mL.

Overall, the mean hospital stay was 6.9 days (range 1–53 days); in patients with positive SARS-CoV-2 specific IgM or IgG, the mean hospital stay was 10.2 days (range 4–33). One patient, with negative tests for SARS-CoV-2, died a few hours after admission due to septic shock due to bowel infarction. All the patients were submitted to outpatient follow-up, consisting in clinical visit and, if required, blood tests. At one-month follow-up, we observed surgical-related complications in 10 (11.6%) patients: we observed 4 cases of hypoparathyroidism following total thyroidectomy, 2 cases of wound seroma after axillary dissection for breast cancer and 2 after incisional hernia repair, and 1 case of surgical site infection following Hartmann's operation for complicated acute diverticulitis. For the purpose of the study, one month after discharge all the patients were contacted, and a questionnaire was administered to evaluate if symptoms SARS-CoV-2 like had developed after the discharge. Two patients developed fever, cough and asthenia, two and three weeks after discharge, respectively; both were managed from their own general practitioner and underwent nasopharyngeal swab, that was positive for SARS-CoV-2 infection in one case; epidemiological investigations demonstrated that the infection was contracted after hospital discharge after a contact with a confirmed case.

The main limitation of this study is that it is a monocentric study, thus, also considering the high variability of the epidemiological distribution of the infection of SARS-CoV-2, it can't be considered representative of a large population.

However, we think that some global reflections could be made.

Considering: a) the current limitations in sensitivity of nasopharyngeal swab, that is about 70% in symptomatic patients and in dedicated Centers.

b) the current areas of uncertainty in the natural history of SARS-CoV-2, including the duration of infection, the rate of viral transmission at seroconversion and the possible presence of long-term -even totally asymptomatic-viral spreaders.

c) the here reported data during the first trimester of CoV-2 among unselected surgical patients spread that estimate the prevalence of SARS-CoV-2 to be higher than the general population (5.8% vs 2%).

Taking into account these data, we would urge to continue providing and to better plan careful preadmission triage and tests, the use of personal protective equipment also in triage, as inconsistent use of one (or more) between goggles, gowns, gloves, and caps may be associated with a higher risk for SARS-COv-2 infection in case of contact with an infected patient, and safe management of surgical smoke, that we think are mandatory also in the setting of non-dedicated COVID-19 hospital in a low CoV-2 incidence area [4,5]. Furthermore, given the pitfalls of clinical assessment to diagnose SARS-Cov-2 infection, the timing to have the results of antibody and RT-PCR is a critical issue and should be improved in each setting.

## Ethical approval

Ethical approval was not needed for this study.

## Sources of funding

No funding for the research was received.

## Author contribution

Fabio Medas: conception and design of the study, acquisition of data, analysis and interpretation of data, drafting the manuscript, final approval of the version to be published. Federico Cappellacci: design of the study, acquisition of data, analysis of data, drafting the manuscript, final approval of the version to be published. Giacomo Anedda: design of the study, analysis and interpretation of data, critical revision of the manuscript, final approval of the version to be published. Gian Luigi Canu: Interpretation of data, critical revision of the manuscript, final approval of the version to be published. Stefano del Giacco: Conception of the study, interpretation of data, critical revision of the manuscript, final approval of the version to be published. Pietro Giorgio Calò: Conception of the study, interpretation of data, critical revision of the manuscript, final approval of the version to be published. Davide Firinu: conception and design of the study, interpretation of data, drafting the manuscript, final approval of the version to be published.

## Trial registry number

1. Name of the registry: ClinicalTrials.gov.

2. Unique Identifying number or registration ID: NCT04480580.

3. Hyperlink to your specific registration (must be publicly accessible and will be checked): https://register.clinicaltrials.gov/prs/app/action/SelectProtocol?sid=S000A2UO&selectaction=Edit&uid=U0004HL5&ts=2&cx=sakyte.

## Guarantor

Dott. Fabio Medas.

## Consent

Informed consent was administered to each patient involved in the study.

## Provenance and peer review

Not commissioned, externally peer-reviewed.

## Declaration of competing interest

The authors declare no conflicts of interest.
